# Harnessing the power of theorising in implementation science

**DOI:** 10.1186/s13012-019-0957-4

**Published:** 2019-12-11

**Authors:** Roman Kislov, Catherine Pope, Graham P. Martin, Paul M. Wilson

**Affiliations:** 1 Room 5.24 Business School, Manchester Metropolitan University, Oxford Road, Manchester, M15 6BH UK; 2 University of Manchester, Manchester, UK; 3 University of Oxford, Oxford, UK; 4 University of Cambridge, Cambridge, UK

**Keywords:** Theorising, Mid-range theory, Programme theory, Grand theory, Implementation science, Research agenda, Theoretically informative research, Mechanism-based explanation, Interdisciplinarity, Circle of enquiry

## Abstract

Theories occupy different positions in the scientific circle of enquiry as they vary in scope, abstraction, and complexity. Mid-range theories play a crucial bridging role between raw empirical observations and all-encompassing grand-theoretical schemes. A shift of perspective from ‘theories’ as products to ‘theorising’ as a process can enable empirical researchers to capitalise on the two-way relationships between empirical data and different levels of theory and contribute to the advancement of knowledge. This can be facilitated by embracing theoretically informative (in addition to merely theoretically informed) research, developing mechanism-based explanations, and broadening the repertoire of grand-theoretical orientations.

## Background

The last few decades have seen a rapid accumulation, systematisation, and advancement of knowledge about implementation strategies, actors and contexts. This growing empirical knowledge base is increasingly encapsulated in a variety of theories, models, and frameworks. By identifying contextual influences and articulating the mechanisms of implementation, theories can be invaluable for explaining intervention outcomes, predicting how implementation may unfold, and supporting generalisability of research findings across a range of settings [1]. Well-developed theory ‘enables knowledge to emerge out of seeming chaos’, providing a common language for studying implementation phenomena and guiding the actual practice of implementation [2]. The gradual maturation of implementation science as a discipline is also reflected in laudable endeavours to systematise and make sense of this theoretical knowledge [3–6] as well as to critically reflect on the current state of the field in general [7, 8].

This editorial contributes to this agenda by suggesting a number of directions for further advancement of theoretical knowledge in implementation research. Our argument builds on a number of observations. First, implementation science is an inherently applied field of inquiry, whose theoretical base is important in guiding knowledge translation and achieving positive impact on the outcomes of implementation strategies. Second, implementation science is an inherently interdisciplinary field that derives and integrates theoretical insights from a number of well-established social science disciplines, such as psychology, sociology, economics, and organisation studies, providing tools for studying implementation at different levels of analysis. Finally, the theoretical base of implementation science is developing in line with the increasing complexity and variability of implementation interventions that unfold in diverse and changing contexts [9]. It is therefore imperative that we cumulatively build theoretical knowledge that is empirically grounded, firmly embedded in broader social science, and flexible enough to accommodate new developments.

We argue that achieving these aims can be facilitated by considering mid-range theories of implementation within the broader scientific circle of enquiry which brings together empirical data and theories at different levels of abstraction. We call for the shift of focus from ‘theory’ as a relatively isolated, static, reified source guiding implementation, towards embracing ‘theorising’ as a set of processes that aim to use empirical data actively in developing, validating, modifying, and advancing conceptual knowledge in the field. More specifically, we suggest three directions for harnessing the power of theorising in implementation science: (1) approaching empirical data in a theoretically informative way; (2) theorising the dynamic relationships between interventions, implementers, and contexts through mechanism-based explanations; and (3) broadening the repertoire of major theoretical traditions derived from other disciplines to inform mid-range theorising. Our suggestions may be of use to authors seeking to publish in *Implementation Science* as they are expected to clearly articulate how their empirical work adds to the existing theoretical thinking in the field [10].

## Mid-range theories in the scientific circle of enquiry

In the social sciences, theory can be broadly defined as ‘an ordered set of assertions about a generic behaviour or structure assumed to hold throughout a significantly broad range of specific instances’ [11]. By developing concepts and explicating their interrelationships, it also seeks to postulate how and why a phenomenon occurs [12]. Theories, however, differ widely by the degree to which their generalisations are ordered, by the level of abstraction at which they explore social phenomena, and by the range of ‘specific instances’ to which they apply. It is therefore possible to distinguish between the following progressively higher levels of conceptual framing: *programme*, or *small*, *theories* that pertain to specific interventions, *mid-range theories* whose application is restricted to a certain subset of social phenomena relevant to a particular range of contexts, and *grand theories*, aiming to construct all-encompassing meta-narratives that span space and time (Table 1) [18, 21].

**Table 1:** Levels of theory in the social sciences

	Definition	Characteristics	Types and examples
Grand theories	All-inclusive systematic efforts to develop a master conceptual scheme, often aspiring to present a unified theory of the social world	- Formulated at a high level of abstraction, often without an underlying empirical base - Non-specific and may lack clear operational definitions of key concepts - Often loosely knit and internally diversified - Less amenable to empirical testing; sometimes unfalsifiable	- Overarching theoretical perspectives through which one sees and interprets the world (e.g. feminist theory and critical theory) - Theoretical oeuvres of sociological classics (e.g. Bourdieu, Giddens, and Marx)
Mid-range theories	Theories that lie between the working hypotheses that evolve in abundance during day-to-day research and the all-encompassing speculations comprising a master conceptual scheme	- Delimited in their area of application - Demonstrate strong interdependence with empirical observations - Specify mechanisms, i.e. social processes having designated consequences for designated parts of the social structure - Not usually derived from grand theories but are often influenced by or consistent with one or several of them	- *Lower-order:* theories aggregating individual programme theories of similar interventions [13] - *Core* implementation science theories (e.g. Normalisation Process Theory [14] and i-PARIHS [Integrated Promoting Action on Research Implementation in Health Services] framework [15]) - *Higher order:* consolidating frameworks combining a number of constructs from pre-existing mid-range theories (e.g. CFIR [Consolidated Framework for Implementation Research] [16] or TDF [Theoretical Domains Framework] [17])
Programme theories	‘Small theories’ providing a sensible and plausible explanation about how a specific policy, intervention, or project is supposed to function and achieve its objectives	- Purposefully practical and accessible, providing concrete working models rather than higher-level abstractions - Uncover assumptions about the mechanisms linking the intervention’s inputs, components, and processes to its outcomes - Involve informal elements representing the perspectives of intervention stakeholders - Usually provisional and subject to modification in the course of an intervention	- Programme theories of individual implementation and improvement projects [18] - Programme theories of large-scale and composite knowledge translation initiatives, such as the National Institute for Health Research Collaborations for Leadership in Applied Health Research and Care (NIHR CLAHRCs) [19] or the Quality Enhancement Research Initiative (QUIERI) [20]

Mid-range theories are seen as fundamental for all social sciences as they are sufficiently broad to provide practically adequate explanations applicable to a range of contexts, yet focused enough to generate testable propositions and guide empirical enquiry [11, 22]. Implementation science, with its well-delineated scope, applied nature, and strong emphasis on the interdependence of theory and data, is no exception. Mid-range theories play an important bridging role between empirical observations (and programme theories based on them), characterised by a low level of abstraction and generalisability, and the highly generic and abstract ‘view from on high’ [23] offered by grand theories. Boundaries between different levels of theorising are not always clearly delineated: a highly systematised programme theory conceptualising an intervention across multiple settings can be viewed as a lower-order mid-range theory [13], whilst those implementation frameworks consolidating multiple pre-existing theories aim to present a more generic view of implementation and can thus be viewed as ‘mid-range theories of higher-order’ positioned closer to grand theories in the conceptual ‘ladder’ [24]. (As we note below, though, they can also risk accumulating and cataloguing constructs without offering additional analytical purchase.)

**Fig. 1:**
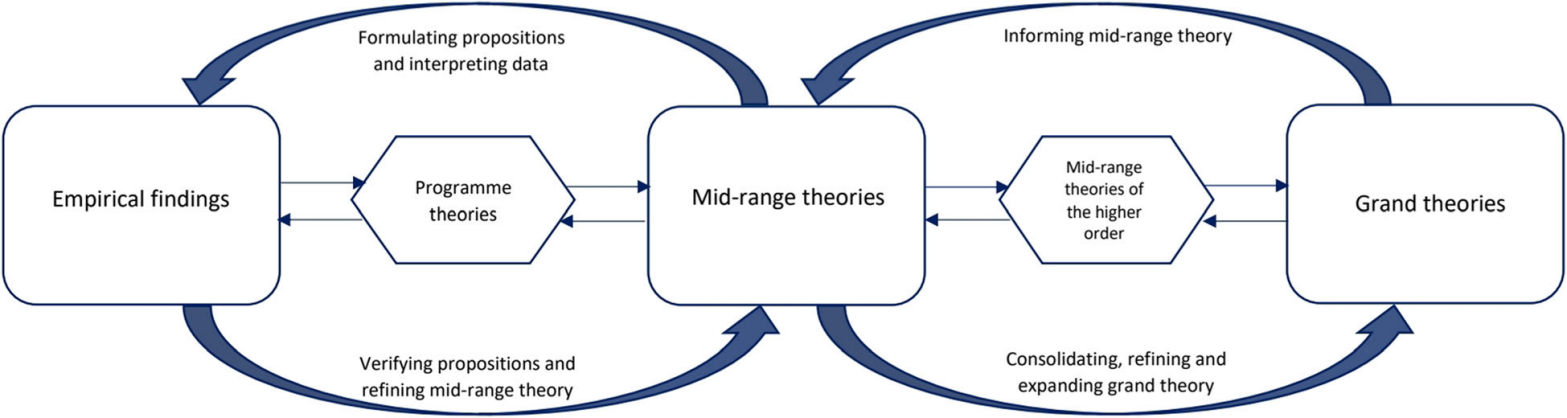
Bridging role of mid-range theories in the scientific circle of enquiry (Adapted from Brodie et al. [25])

The bridging role of mid-range theories can be demonstrated in the ‘scientific circle of enquiry’ [25] emphasising the two-way connections between empirical observations and theories at different levels of conceptual abstraction (Fig. 1). Mid-range theories generate testable propositions which can, in turn, be informed by grand theories. Empirical findings are then used to modify the premises of mid-range theories, thus refining and expanding their scope. These modified mid-range theories can then be consolidated into higher-order theoretical perspectives, potentially refining and expanding the scope of grand theories. It is this intermediate role of mid-range theories that makes their development crucial for the advancement of the health and social sciences [22].

We argue that the ability of implementation science to explain and guide implementation can be enhanced by capitalising on the bridging role of mid-range-theories, acknowledging the inherently iterative and fluid nature of theoretical work, and paying greater attention to the two-way connections between different elements of the scientific circle of enquiry. This mandates a change of perspective from ‘theories’ as finished products to ‘theorising’ as the process of developing, refining, and expanding theoretical knowledge [26, 27]. We follow Weick in acknowledging that products of theorising seldom emerge as fully developed theories, resulting instead in ‘approximations’ or ‘interim struggles’ that can inform subsequent work and thus contribute to incremental accumulation of knowledge [28]. In what follows, we offer a set of directions for fruitful engagement with theorising and moving the discipline forward.

## Theoretically informative implementation research: using empirical data to refine theory

Although use of theory in implementation science has increased over time [29], research to date has adopted a largely *theoretically informed* approach, where theory is applied to design an intervention or to systematise or explain process evaluation findings [1]. Ironically, many of the theories, models, and frameworks used to guide implementation research and practice were not themselves the product of rigorously collected and analysed empirical data. Nevertheless, rather than scrutinising theoretical assumptions in the light of empirical findings, implementation researchers tend to treat theoretical knowledge as ‘received wisdom’ to be applied with reverence rather than challenged, developed, and moved forward. For instance, a systematic review on the use of normalisation process theory (NPT) shows that studies informed by this theoretical approach rarely engage in its critique or add a contribution to it [30]. Overall, engagement with theory in implementation research often remains one-way, with theory shaping data collection and analysis, but little effort being made to explain what the resulting empirical findings mean for the development of that theory [1]. Correspondingly, theories become reified or ossified. They can also become scripted accounts that offer a go-to explanation for any observed social phenomenon, plucked off the shelf without thought or reflection.

We call for a broader utilisation of *theoretically informative* empirical research which seeks to yield new theoretical insights applicable to a wide range of settings. Such research, aimed at developing ‘theory-building implementation science’ [31], is premised on a constant dialogue between the theoretical and the empirical. Theory guides empirical enquiry, enabling the researcher to see things in the data that might otherwise be taken for granted and develop new theoretical hypotheses or propositions that are, in turn, evaluated by empirical observations [32]. In critical realist trials, which bring together outcome and process data collected in a theoretically informed way across several stages, iterative refinement, augmentation, and testing of study hypotheses can lead to the development of empirically informed mid-range theory [33, 34]. In qualitative small-sample studies, a *particular* empirical case (or set of cases) can be used for further refining existing theoretical conceptualisations of the *general* processes [35]. Here, theory is a tool which should be improved with each subsequent application, rather than merely having its utility confirmed [36]. Theorising becomes an iterative and recursive process [30, 35]: theory is no longer seen as ‘fixed and immutable’—a holy text to be corrupted at one’s peril—but as ‘a fluid collection of principles and hypotheses’ [37]. Interestingly, this dynamic approach is apparent in the development of NPT and PARIHS, both of which have evolved significantly in response to empirical verification and conceptual critique [15, 30].

Adopting a theoretically informative approach would require important changes in how implementation scientists approach research design [1]. First, an empirical case under investigation should be positioned against, and compared with, previous studies that have contributed to the formulation and development of the relevant theory. Rather than rigidly adhering to the original theoretical account, an emphasis should be placed on creatively synthesising previous knowledge in ways that illuminate the real-world implementation issue to be explained. Second, analysis and interpretation of findings should not be limited to identifying similarities between the empirical case and extant theory, but should aim to identify the differences and/or omissions, express them in theoretical terms, and use these newly identified variations to refine previous theoretical knowledge. These insights do not have to be large-scale and transformative to be revelatory and original [12], though they do need to represent more than tinkering, navel-gazing, or adding terminological clutter—potential side effects observed when ‘theoretical contribution’ is valorised and risks becoming an end in itself [38, 39]. Finally, when undertaking data analysis, it is important to avoid producing ‘shopping lists’ of themes that are purely descriptive or simply catalogue multiple contextual factors or processes of change. Themes and propositions should evolve from the data that link different determinants, concepts, or factors together, thus reflecting relationships between them [40]. This is discussed in more detail in the next section.

## Mechanism-based explanations: theorising dynamic relationships between interventions, implementers, and contexts

Recent years have seen the proliferation of process models and determinant frameworks [2, 3]. *Process models* present an ideal view of implementation and prescribe steps or stages that need to be executed for accomplishing implementation goals. *Determinant frameworks*, or ‘static theories’ [41], focus on identifying and cataloguing multiple (and often heterogeneous) components of healthcare systems which act as ‘barriers’ or ‘enablers’ to successful implementation, thus influencing its outcomes. Frameworks of this kind can alert researchers to the range of components, at multiple levels of social reality, that should be accounted for in intervention design and evaluation. They can be useful for explaining variation in observed outcomes in retrospect or predicting them a priori [2]. However, determinant frameworks tend to focus on assembling these components in a number of higher-order ‘domains’ that are often preoccupied with the ‘anatomy’ of implementation rather than its ‘physiology’ [41], with some critics referring to them as ‘structured lists of disconnected items’ [7]. Overall, process models and determinant frameworks can be considered rudimentary and implicit forms of theory, often reducing complex relationships to prescriptive checklists or stages. Relatively little attention is paid to explicating functional relationships between different determinants, causal mechanisms through which different stages of implementation or contextual variables influence outcomes, or additional mediators and moderators affecting these causal pathways [42]. This is accompanied, perhaps unsurprisingly, by a relative paucity of theory testing and refinement in empirical research informed by these models and frameworks [2].

Applying a theoretically informative approach to existing frameworks could address some of these shortcomings and lead to developing critical, relational, and dynamic approaches to theorising the complex interplay between the characteristics of interventions, the activities of implementers, and the properties of variable broader contexts [9, 43]. The essence of theorising lies in its ability to uncover generative mechanisms of social phenomena, and implementation research can make an important contribution by detailing the ‘cogs and wheels’ of the causal processes through which implementation outcomes are brought about [42]. Mechanism-based explanations are selective: rather than embellishing existing implementation frameworks with even more exhaustive or forensically dissected sets of factors, it may be more beneficial to focus on a relatively limited number of elements relevant to the problem at hand, and to explore complex relationships and interdependencies between them in depth [1, 44]. Rather than treating mechanisms as intervening variables, a mechanism-based explanation discloses their internal structure, shedding light onto ‘how the participating entities and their properties, activities, and relations produce the effect of interest’ [44]. Mechanisms can involve a range of more dynamic processes, including multiplication, non-linear relationships, feedback loops, and phase transitions, and mid-range theorising can further explore the resulting evolution of structures and practices triggered by implementation interventions [43].

Developing this agenda will involve several important shifts. First, when conceptualising the fidelity of implementation interventions, the focus should move away from the precise ‘form’ of an intervention (i.e. what is being delivered) towards its ‘functions’ (i.e. what processes are initiated) and ‘purposes’ (i.e. through which mechanisms of change intervention components work) [34, 45]. As shown, for example, by studies of facilitation as an implementation strategy [46, 47], intervention integrity should be defined functionally in relation to fit with the underlying causal mechanisms (what the intervention *does*), rather than compositionally (what the intervention *is*). Second, given that context is ‘a *process* rather than a *place*’ [9], flexible longitudinal designs are needed to verify existing process models and explore the emergent and dynamic aspects of implementation. Adopting a temporal perspective would also enable us to switch from a current preoccupation with the beginnings of implementation journeys towards enhancing our understanding of sustainability and scale up [9]. Finally, more attention is required to explore the experiences of, and relationships within and between, different groups (such as policymakers, managers, researchers, clinician, and patients) involved in the processes of design, implementation, spread, and scale up of interventions, as well as the potential effects of these experiences and relationships on intervention outcomes.

## Pluralism and diversity: broadening the repertoire of grand-theoretical orientations

Mid-range implementation theories have been shaped by major theoretical orientations derived from other disciplines. The evidence-based practice paradigm [48] and the discipline of behaviour change psychology [17] have been particularly influential in this regard, whilst the iterative development of NPT has involved continuous engagement with fundamental theoretical questions debated by several sociological schools of thought [14, 30]. The theoretical basis of implementation science is thus clearly interdisciplinary, but this interdisciplinarity does not necessarily channel down to the level of empirical exploration, where cross-fertilisation with other social science disciplines and their theoretical orientations remains relatively low and somewhat unequal [31, 49, 50]. Theoretical ideas imported from other fields still tend to be subjected to predominantly deductive, determinant-focused styles of thinking. Traditions dealing with group-level, organisational and systemic levels of analysis tend to be less utilised in research and practice than individual educational and psychological approaches [7, 43, 49]. This may result in the lack of concordance between the types of implementation problem identified and the approaches to change chosen to address them, which is further aggravated by the fact that implementation researchers and practitioners may be ‘stubbornly consistent’ in sticking to their preferred methodological orientations [7]. Conversely, as an emergent field at the intersection between multiple disciplines, many of those who engage in implementation science are disciplinary ‘agnostics’ who lack in-depth training in core social science disciplines and have a relatively limited theoretical repertoire to draw on in explicating empirical findings.

We believe that these issues could be addressed both by exposing implementation researchers to a variety of theoretical and disciplinary traditions that have already entered the toolbox of implementation science and by opening up to new perspectives. Diversity of philosophical and theoretical approaches, accumulated by the social sciences, genuinely reflects the complexity of the social world and the multiple ways we can make sense of it [51, 52]. Table 2, drawing on the work of Patton [51], provides examples of grand-theoretical traditions that could be successfully deployed by implementation researchers to address various questions and thus broaden the repertoire of implementation science. Engaging with diverse styles of theorising has the potential to uncover complex and processual forms of causality, where constructs interact in bidirectional, cumulative, or emergent ways, and to cut across multiple levels of analysis [53]. It may also stimulate fruitful exploration of those issues, such as gender, power, and equality, that have so far received little explicit theoretical attention in implementation science. At the same time, it should be kept in mind that empirical studies relying on grand theories may be at risk of becoming absorbed in the pre-existing all-encompassing master schemes offered by these theories, failing to develop distinctive new ideas and instead merely reproducing prior theoretical understanding. This further underscores the importance of mid-range theorising that can selectively apply, operationalise, and refine the assumptions of grand theories—which by no means should be immune from the theoretically informative approach described above—by subjecting them to empirical verification [23, 27]. (See, for example, an empirical study selectively deploying Bourdieu’s concepts and ideas to develop a mid-range theory of boundary spanners’ legitimacy [54].)

**Table 2:** Grand-theoretical traditions and their potential relevance to implementation science (adapted from Patton [51])

Perspective	Disciplinary roots	Central questions relevant to implementation science
Ethnography	Anthropology	What is the culture of a certain group of people (e.g. an organisation) involved in implementation? How does it manifest in the process of implementation?
Critical realism	Philosophy, social sciences and evaluation	What are plausible explanations for verifiable patterns of implementation?
Constructivism	Sociology	What are the implementation actors’ reported perceptions, explanations, beliefs, and worldviews? What consequences do these have on implementation?
Phenomenology	Philosophy	What is the meaning, structure, and essence of the lived experience of implementation for a certain group of people?
Symbolic interactionism	Social psychology	What common set of symbols and understandings has emerged to give meaning to people’s interactions in the process of implementation?
Semiotics	Linguistics	How do signs (i.e. words and symbols) carry and convey meaning in particular implementation contexts?
Narrative analysis	Social sciences, literary criticism	What do stories of implementation reveal about implementation actors and contexts?
Complexity theory	Theoretical physics, natural sciences	What is the underlying order of any disorderly implementation phenomena?
Critical theory	Political philosophy	How do the experiences of inequality, injustice, and subjugation shape implementation?
Feminist inquiry	Interdisciplinary	How does the lens of gender shape and affect our understandings and actions in the process of implementation?

Engagement with new theoretical orientations must take into account their underlying philosophical and disciplinary roots rather than merely borrowing concepts haphazardly (and recompiling them in another structured list or static theory). This requires an understanding of the internal logic and assumptions of each approach. If multiple perspectives are combined in one study, the resulting analysis should demonstrate internal coherence, avoid unnecessary complexity and redundancy, and develop novel insights rather than simply ‘repackaging’ what is already known from previous research [6]. It is important to acknowledge and reflect on possible contradictions between the underlying ontological and epistemological assumptions espoused by different approaches [55], but as the somewhat blinkered polemic around realist trials has shown [34, 56, 57], what is understood as commensurable is open to debate, with pragmatic considerations clashing with epistemological purism. However, despite the propensity for contradiction or inconsistency (and accompanying paradigm wars), mutual understanding across different approaches is not only possible but can even be potentially enlightening [52, 58]. We call for co-existence of multiple paradigms in the field of implementation science that would acknowledge the strengths and weaknesses of different forms of explanation, adequately apply them depending on the research question or practical issue at hand, and use sets of assessment criteria appropriate to the philosophical assumptions, theoretical orientations, and methodological approaches deployed [52, 53].

## Conclusion

In this editorial, we have called for *theoretically informative* implementation research. This requires a shift of perspective from ‘theories’ as finished products to ‘theorising’ as an iterative process of advancing knowledge. It is through the verification, refinement, and consolidation of mid-range theories that social science disciplines develop. Engaging with the broad directions for harnessing the power of mid-range theorising described in this article will assist researchers in their efforts to develop new insights and contribute to advancing the knowledge base of implementation science.

## Data Availability

Not applicable.
